# Sustained Action of Imapextide, A Glucagon-Like Peptide-1 Receptor Antagonist, in Healthy Volunteers

**DOI:** 10.1210/clinem/dgaf691

**Published:** 2025-12-24

**Authors:** Elisa Fabbrini, Anastasiya Koshkina, Michael Hackett, Michael Zhao, Kishore Thalluri, Marcus Hompesch, Alejandra Macias, Kristi Schneider, David A D’Alessio, Richard D DiMarchi, Salomon Azoulay

**Affiliations:** MBX Biosciences, Inc., Carmel, IN 46032, USA; MBX Biosciences, Inc., Carmel, IN 46032, USA; MBX Biosciences, Inc., Carmel, IN 46032, USA; MBX Biosciences, Inc., Carmel, IN 46032, USA; Department of Chemistry, Indiana University, Bloomington, IN 47405, USA; ProSciento, Inc., Chula Vista, CA 91911, USA; ProSciento, Inc., Chula Vista, CA 91911, USA; MBX Biosciences, Inc., Carmel, IN 46032, USA; Duke Molecular Physiology Institute, Duke University, Durham, NC 27701, USA; Department of Medicine, Duke University, Durham, NC 27701, USA; Department of Chemistry, Indiana University, Bloomington, IN 47405, USA; MBX Biosciences, Inc., Carmel, IN 46032, USA

**Keywords:** bariatric surgery, clinical trial, GLP-1 receptor antagonist, imapextide, phase 1, postbariatric hypoglycemia

## Abstract

**Context:**

After weight-loss surgery, some patients experience postbariatric hypoglycemia (PBH). Although PBH is customarily managed by dietary modifications with continuous glucose monitoring, advanced treatment options are needed. Imapextide is a sustained-action, selective, reversible glucagon-like peptide-1 (GLP-1) receptor antagonist designed for once-weekly administration for PBH treatment.

**Objective:**

This work aimed to evaluate safety, tolerability, pharmacokinetics (PK), and pharmacodynamics (PD) of single and multiple ascending doses (SAD/MAD) of imapextide in healthy volunteers (HVs).

**Methods:**

This 3-part, phase 1, double-blind study enrolled healthy adults. In the SAD/MAD parts, randomly assigned participants (3:1) received subcutaneous imapextide (SAD, 10-200 mg; MAD, 10-30 mg once weekly, 4 doses) or placebo. The primary end point was safety/tolerability (treatment-emergent adverse events [TEAEs]). PK (SAD/MAD; time-to-maximal concentration [t_max_] and half-life [t_1/2_]) and PD (MAD; GLP-1 and glycemic parameters during a mixed meal tolerance test [MMTT]) were assessed. Imapextide drug-drug interactions (DDIs) of OATP1B1/OATP1B3 inhibition and accelerated gastric emptying were evaluated with coproporphyrin-I, rosuvastatin, and acetaminophen.

**Results:**

Overall, 69 HVs were randomly assigned (32 SAD, 23 MAD, 14 DDIs). Imapextide TEAE rates ranged from 33.3% to 50.0% (SAD), 33.3% to 100% (MAD), and 14.3% (DDIs) across cohorts. The most common TEAEs included injection site erythema (SAD, MAD) and injection site swelling (MAD). Imapextide exposure was dose proportional (median t_max_ 24-48 hours; mean t_1/2_ ∼90 hours). During the MMTT, imapextide increased GLP-1 concentrations at early time points, while effects on other glycemic parameters were variable. Minimal nonclinically relevant DDIs occurred with rosuvastatin. Imapextide slightly accelerated gastric emptying.

**Conclusion:**

Imapextide was generally well tolerated in HVs. PK findings supported weekly dosing. PD showed consistent effects on GLP-1 levels, supporting a potential role for imapextide in modulating PBH pathophysiology.

Bariatric surgery is a highly effective and sustainable treatment for obesity and weight-related comorbidities (1-4). The number of bariatric surgeries performed globally has increased over the past decade; the total number of metabolic and bariatric surgeries performed worldwide was approximately 500 000 in 2020 and 600 000 in 2021, of which approximately 180 000 were conducted in North America (5). Despite the clinical effectiveness of bariatric surgery, some patients experience complications, such as postbariatric hypoglycemia (PBH) (1, 2, 4, 6). Anatomical restructuring of the gastrointestinal (GI) tract from bariatric surgery, primarily Roux-en-Y-bypass of the pylorus and proximal intestine, leads to rapid transit of nutrients into the intestine after eating, abrupt supraphysiologic increases in blood glucose, excessive glucagon-like peptide 1 (GLP-1) responses, and consequent hypersecretion of insulin; these postprandial hyperinsulinemic hypoglycemic effects are exaggerated in patients with PBH (1, 7). PBH symptoms typically manifest between 1 and 3 hours after meals (particularly high-carbohydrate meals) and include neuroglycopenic and adrenergic symptoms, such as dizziness, impaired cognition, palpitations, lightheadedness, sweating, confusion, falls, seizures, and loss of consciousness (2-4, 6, 8). PBH episodes can occur nocturnally if food is ingested late in the day, suggesting the importance of treatments with sustained duration of biological action (9). Symptoms of PBH can significantly reduce patients’ quality of life and impair daily activities, and more severe cases of PBH can be life-threatening (2, 8). PBH is considered a rare condition and is challenging to diagnose (often confused with dumping syndrome); there is substantial variability in clinical presentation with severity of symptoms ranging widely, and estimates of prevalence dependent on the diagnostic approach (1, 3). It is very likely that PBH is underdiagnosed and underreported (1, 3, 4, 6).

Treatment options for PBH are limited, and there are no currently approved treatments (4). Current guidelines to manage symptoms of PBH recommend dietary modification, such as small meals and avoidance of simple and high-density carbohydrates, as the cornerstone of PBH treatment, and this is used in conjunction with continuous glucose monitoring (3, 4, 6, 8, 10, 11). A number of medical treatments have been tried off-label, including acarbose, diazoxide, somatostatin analogues, canagliflozin, or dasiglucagon (3, 4, 6, 8, 12, 13). However, success with these agents is variable and they rarely provide a significant remedy of symptoms. There are no approved treatments for PBH, and the need for new treatments is critical in this patient population that currently has limited therapeutic options.

PBH has been described in patients with the 2 most common bariatric surgical procedures, Roux-en-Y gastric bypass and sleeve gastrectomy, although the syndrome is much more common in gastric bypass (2). Because of the increased secretion of GLP-1 after meals in people with gastric bypass, the GLP-1 pathway has been studied as a target of therapy. Several studies have demonstrated that GLP-1 receptor antagonists prevent prandial hypoglycemia in people with gastric bypass, and several investigational therapies have been advanced with the goal of reducing postprandial insulin levels by inhibiting GLP-1 signaling (2, 8, 14). Results from clinical studies of GLP-1 antagonists such as avexitide have shown clinical benefits of GLP-1 inhibition in patients with PBH (14-17). However, the short half-life of avexitide (t_1/2_; ∼3.5 hours) (16, 17) requires daily injections to maintain daytime exposures in the efficacious range and limits its exposure to target tissues during the evening and night. In addition, daily injections are often burdensome for patients, thus undermining long-term treatment adherence and compliance.

Imapextide (previously referred to as MBX 1416) is a selective, reversible GLP-1 receptor antagonist developed to treat PBH. Imapextide was designed to counteract excessive postprandial GLP-1 secretion, preventing postprandial hyperinsulinemic hypoglycemia, and mitigating the limiting neuroglycopenic symptoms suffered by patients with PBH. Imapextide was developed to provide dose-proportional active drug with sustained time of action to enable once-weekly administration through consistent and sustained exposure. This provides protection from the effects of excessive GLP-1 secretion at all times, including overnight, through a 1-week period. This first-in-human phase 1 clinical trial evaluated single ascending doses (SAD) and multiple ascending doses (MAD) of imapextide for safety, tolerability, pharmacokinetics (PK), pharmacodynamics (PD), and drug-drug interactions (DDIs) in healthy volunteers (HVs).

## Materials and Methods

### Phase 1 Study Design, Participants, and Treatment

This phase 1, randomized, double-blind, placebo-controlled study (NCT06036784) enrolled healthy adults (aged 18-65 years) with a body mass index of 18 to 30 kg/m^2^, stable body weight for 3 months or longer, and without diabetes. Potential participants were ineligible if they had type 1 or type 2 diabetes or prediabetes (based on medical history, relevant concomitant medications [including oral or injectable antidiabetic medications], fasting plasma glucose concentrations ≥100 mg/dL or glycated hemoglobin concentrations ≥5.7% [American Diabetes Association thresholds for diabetes and prediabetes (18)]). Potential participants were excluded if they had any significant medical history; contraindications; or previous serious adverse reaction, hypersensitivity, or angioedema to GLP-1 receptor agonists, imapextide, rosuvastatin, acetaminophen, or peptide formulation ingredients.

**Figure 1.**
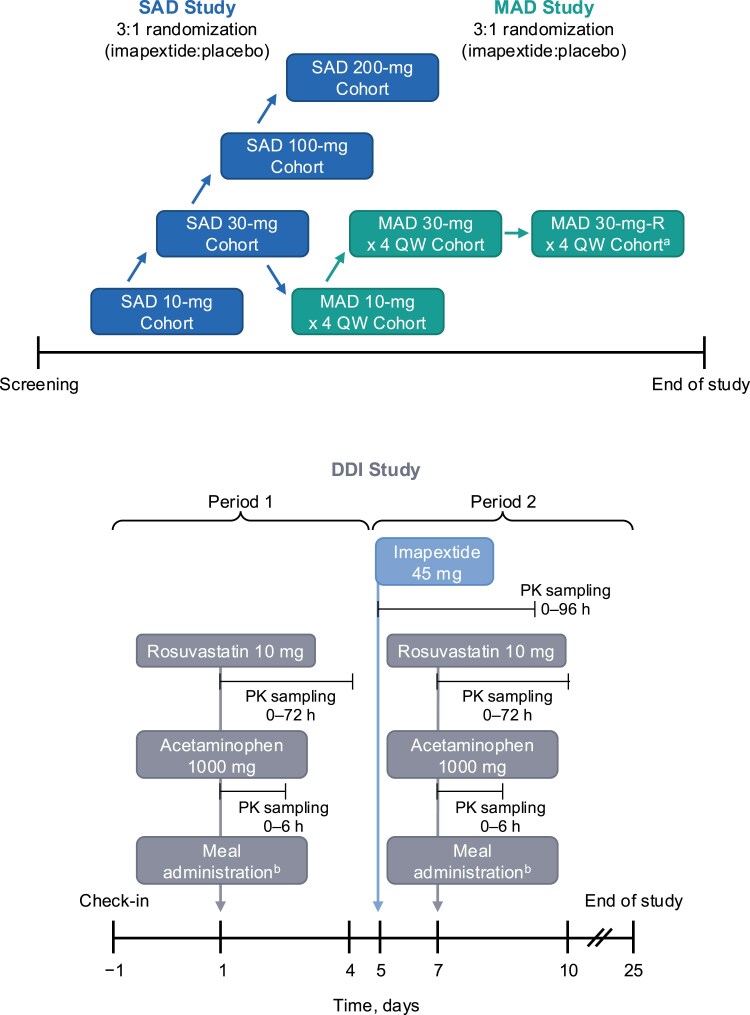
Study design. DDI, drug-drug interaction; MAD, multiple ascending dose; PK, pharmacokinetics; QW, once weekly; SAD, single ascending dose. Studies in the cohorts were performed in sequential order, but overlap may have occurred during the study. ^a^Another cohort received imapextide 30 mg once weekly with medical management of injection site reactions. ^b^Liquid meal of approximately 500 kcal after an overnight fast.

The study comprised 3 parts (SAD, MAD, and DDIs; Fig. 1). Participants remained in a clinical research unit from days −2 to 8 for the SAD cohorts; days −2 to 9, 15 to 16, and 21 to 29 for the MAD cohorts; and days −1 to 10 for the DDI cohort.

The SAD portion of the study included a sequential group design (with a safety review before proceeding to the next dose level) of 4 cohorts, each with 8 HVs randomly assigned 3:1 into 2 treatment arms to receive a single subcutaneously administered dose of imapextide (10-200 mg) or placebo. The primary objective of the SAD portion was to evaluate safety and tolerability. The secondary objective was to characterize the PK profile, and the exploratory objective was to describe the immunogenicity of imapextide.

The MAD portion of the study included 3 cohorts, each with up to 8 HVs randomly assigned 3:1 to receive 4 once-weekly subcutaneously administered doses of imapextide or placebo. One of these cohorts received imapextide at a 10-mg dose. The other 2 cohorts received a dose of imapextide at 30 mg, either split into 2 injections of 15 mg each administered into 2 separate abdominal quadrants (hereafter referred to as the “30-mg cohort”) or as 1 injection (the “30-mg-R cohort”). In the 30-mg-R cohort, participants received treatments (eg, cold compresses, mometasone cream, and loratadine) to prevent and/or reduce erythema at the injection site that characterized injection site reactions (ISRs) observed in the previous MAD cohorts (ie, the 10-mg and 30-mg cohorts). The primary objective of the MAD portion was to evaluate safety and tolerability. The secondary objective was to characterize the PK profile, and the exploratory objectives were to evaluate the immunogenicity of imapextide and the PD effects of imapextide on glycemic parameters during a mixed meal tolerance test (MMTT; described in the PD assessments section).

The DDI portion of the study included 1 cohort of 14 HVs and consisted of 2 study periods. In study period 1 (days 1-5), all participants received rosuvastatin 10 mg (to assess effects on absorption and clearance via OATP1B1/OATP1B3) and acetaminophen 1000 mg (to assess gastric emptying) on day 1. Gastric emptying was evaluated using an acetaminophen absorption test, which measures enteral uptake of orally administered acetaminophen by monitoring serum acetaminophen concentrations over time post dosing; this method has been commonly used to assess gastric emptying in patients receiving GLP-1 receptor agonists (19). Blood samples were also collected to measure clearance of the endogenous biomarker for hepatic OATP inhibition, coproporphyrin-I (CP-I), to assess potential inhibition of OATP1B1/3 by imapextide. In study period 2 (days 5-10), all participants received a single dose of imapextide 45 mg on day 5 (expected to achieve equivalent concentration to the steady state with weekly dosing of 30 mg), followed by rosuvastatin 10 mg and acetaminophen 1000 mg on day 7, concurrent with the anticipated t_max_ of imapextide. The primary objective of the DDI portion was to evaluate the PK of rosuvastatin and acetaminophen in the presence and absence of imapextide. The secondary objective was to evaluate the safety and tolerability of imapextide in the presence and absence of rosuvastatin and acetaminophen, and the exploratory objective was to describe changes in CP-I plasma concentrations.

The study was conducted in accordance with the protocol and International Council for Harmonisation guidelines, as well as applicable regulations, guidelines, and ethical principles originating from the Declaration of Helsinki. The study protocol was reviewed and approved by a local independent ethics committee/institutional review board before the start of the study. All participants provided written informed consent before screening or undergoing study-specific procedures.

### Assessments

#### Safety assessments

The primary objective of the SAD and MAD portions of the study was to evaluate the safety and tolerability of imapextide; safety was also evaluated in the DDI portion. Safety assessments included incidence and severity of treatment-emergent adverse events (TEAEs), changes in physical examination findings, clinical laboratory abnormalities (serum chemistry, hematology, urinalysis, and coagulation; abnormalities were defined as deviation from the laboratory normal range), and potential effect on vital sign measures. All blood samples for safety analyses were collected after an overnight fast (≥8 hours). If present, data on ISRs were collected using a dedicated form and rated on severity according to the US Food and Drug Administration toxicity scale for vaccines (for details on severity grading, see Supplementary Table S1) (20, 21). All ISRs were evaluated, regardless of whether they were reported as TEAEs by the investigator. In the 30-mg-R cohort, participants with ISRs grade 2 or worse underwent a dermatology consultation, including a potential skin biopsy at the discretion of the dermatologist.

#### Pharmacokinetic assessments

A noncompartmental analysis using Phoenix WinNonlin (version 8.0 or higher) was used to evaluate imapextide in the SAD and MAD cohorts. PK parameters included maximum plasma concentration (C_max_), time to C_max_ (t_max_), area under the plasma concentration-time curve (AUC), to the end of the dosing interval (AUC_0-168_), AUC from time 0 extrapolated to infinity (AUC_inf_; SAD only), terminal phase half-life (t_1/2_), and accumulation ratios for AUC and C_max_. In the SAD cohorts, blood samples for bioanalysis were collected pre dose and through 28 days post dose; blood samples for bioanalysis in the MAD cohorts were collected pre dose and through 7 days post dose, with the last sample collected 23 days post dose. Participants fasted overnight (≥8 hours) before blood sampling.

#### Drug-Drug interaction assessments

The DDI portion of the study was conducted to evaluate the effects of imapextide on gastric emptying, which would accelerate the absorption of rosuvastatin and acetaminophen (as well as other enterally administered drugs). Imapextide was also examined as a perpetrator of OATP1B1/3, which would affect the hepatic clearance of rosuvastatin and CP-I. PK assessments for rosuvastatin included C_max_, t_max_, AUC from 0 to 72 hours (AUC_0-72_), and AUC_inf_. PK assessments for CP-I included C_max_, t_max_, AUC_0-72_, and AUC from 0 to 108 hours (AUC_0-108_). PK assessments for acetaminophen included C_max_, t_max_, and AUC from 0 to 1 hour (AUC_0-1_) and 0 to 6 hours (AUC_0-6_). Rosuvastatin and acetaminophen were administered after an overnight fast (≥8 hours), followed by a 500-kcal nutritional shake (Ensure Plus, 360 g, Abbott). Blood samples were drawn pre dose and post dose for bioanalysis of imapextide (pre dose on day 1/period 2 only; post dose at 12, 24, 36, 48, 60, 72, and 96 hours), rosuvastatin (post dose at 0.5, 1, 1.5, 2, 3, 4, 6, 8, 10, 12, 14, 24, 36, 48, and 72 hours), and acetaminophen (post dose at 30, 60, 90, 120, 180, 240, 300, and 360 minutes).

#### Pharmacodynamic assessments

PD assessments during the MAD portion of the study included absolute levels and placebo-adjusted change from baseline in plasma glucose, insulin, C-peptide, gastric inhibitory peptide (GIP), peptide YY (PYY), glucagon, total GLP-1, and active GLP-1 concentrations during an MMTT. A standardized 300-minute MMTT was conducted on study days −1, 9 (day of assumed C_max_ for the second dose of imapextide), and 23 (day of assumed C_max_ for the fourth and last dose of imapextide) using a 700-kcal nutritional shake (Ensure Plus, 16 fluid ounces [∼473 mL], Abbott) to provide consistent nutrient consumption across the 3 MMTTs. Following an overnight fast (≥8 hours), blood samples for PD assessments were collected before and through 5 hours after initiation of the MMTT. Blood samples for plasma glucose were drawn at 30, 15, and 0 minutes before the mixed meal and 10, 20, 30, 40, 50, 60, 75, 90, 105, 120, 135, 150, 165, 180, 210, 240, 270, and 300 minutes after initiation of the mixed meal. Blood samples for insulin, C-peptide, GIP, PYY, glucagon, and GLP-1 were drawn 30, 15, and 0 minutes before and 15, 30, 45, 60, 90, 120, 180, 240, and 300 minutes after initiation of the mixed meal. Enzyme-linked immunosorbent assays (Mercodia) were used to measure total GLP-1 (catalog No. 10-1278-01, lot No. 35652, RRID: AB_2892202), total GIP (catalog No. 10-1258-01, Lot #35492, RRID: AB_2895085), and glucagon levels (catalog No. 10-1271-01, lot No. 35843, RRID: AB_2737304). V-PLEX Plus Metabolic Panel 1 kits (Meso Scale Discovery) were used to measure active GLP-1 and active GIP levels (catalog No. K15325D, lot No. D0082071, RRID: AB_3683506). U-PLEX Human PYY (total) Antibody Set (Meso Scale Discovery) was used to measure PYY levels (catalog No. B216B-Series, lot No. SET02387A, RRID: AB_3717572). C-peptide and insulin levels were measured by LabCorp using electrochemiluminescence immunoassays (C-peptide, LabCorp test No. 010108; insulin, LabCorp test No. 004333).

#### Immunogenicity

Immunogenicity was assessed by measuring the presence of antidrug antibodies (ADAs) in plasma before and after study drug administration (on days −2, 8, 15, 22, 29, 36, and 45, following an overnight fast) using a standard multitier assay approach to detect, confirm, and measure the titer of reactive antibodies.

### Statistical Analysis

In the in vitro analyses, luminescence data were plotted using Origin 2019b software (OriginLab) and the half-maximal effective concentration (EC_50_) or half-maximal inhibitory concentration (IC_50_) was determined by logistic fitting. Potency was determined by comparative analysis of relative EC_50_ or IC_50_ values.

Participants who received placebo were pooled for all SAD cohorts and all MAD cohorts for SAD and MAD analyses, respectively. A formal sample-size calculation was not performed as this descriptive analysis focused on assessing safety and tolerability across several imapextide dose levels in the SAD and MAD groups. For each study portion, a sample size of 8 participants in each cohort (6 active, 2 placebo) was considered appropriate for the evaluation of safety, tolerability, and PK data; 6 participants taking the active drug provided a probability of 80% or greater of observing at least 1 occurrence of any AE with a true incidence rate of 24% or greater. A formal test to assess the normality of variables was not conducted, given the limited sample size and lack of statistical power. A parametric analysis was performed to assess the increment of active GLP-1 levels from baseline. A *t* test was used to compare placebo-adjusted change from baseline in PD parameters during an MMTT at day 9 and day 23. All other results were descriptively summarized with no inferential statistical comparisons between treatment groups. Statistical analyses were conducted using SAS version 9.4 or higher. Safety analyses were conducted among all participants who received 1 or more doses of the study drug; PK and PD results are reported as observed cases with no imputation for missing data.

## Results

### Phase 1 Study Participants

**Figure 2.**
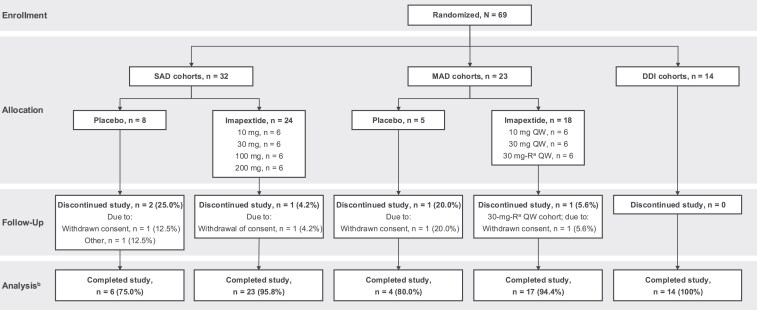
Participant disposition. DDI, drug-drug interaction; MAD, multiple ascending dose; QW, once weekly; SAD, single ascending dose. ^a^8 participants were planned for the MAD 30-mg cohort, but only 7 were enrolled (imapextide 30 mg n = 6; placebo n = 1). The eighth potential participant would have been randomly assigned to the placebo group; thus, only 5 participants were enrolled in the MAD placebo group (2 participants each randomly assigned to placebo in the 10-mg and 30-mg-R cohorts, and 1 participant in the 30-mg cohort) rather than the planned 6 participants. ^b^Another cohort received imapextide 30 mg once weekly with medical management of injection site reactions. ^b^Safety analyses were conducted among all participants who received 1 or more doses of study drug; pharmacokinetics and pharmacodynamics analyses were conducted using observed values available at each study visit with no imputation for missing data.

A total of 69 HVs were enrolled in the study: 32 participants in the SAD cohorts (placebo, n = 8; imapextide, n = 24), 23 in the MAD cohorts (placebo, n = 5; imapextide, n = 18), and 14 in the DDI cohort (Fig. 2). Five participants discontinued the study (2 in the SAD placebo group, 1 in the SAD imapextide 10-mg group, 1 in the MAD placebo group, and 1 in the MAD imapextide 30-mg-R group); all discontinuations were due to personal reasons (eg, withdrawn consent); none were safety related. Most participants in all 3 portions of the study were White, but racial distribution in the SAD and MAD cohorts varied across treatment groups; mean age and body weight were lowest in the imapextide 100-mg group (Table 1). The proportions of male and female participants were generally balanced across treatment groups in the SAD cohorts, while most participants in the MAD and DDI cohorts were male.

**Table 1.** Baseline demographics and characteristics in the single ascending dose, multiple ascending doses, and drug-drug interaction cohorts

SAD cohorts					
Characteristic	Placebo n = 8	Imapextide			
		10 mg n = 6	30 mg n = 6	100 mg n = 6	200 mg n = 6
Age, mean (SD), y	44.4 (7.7)	36.3 (6.0)	51.8 (10.6)	35.7 (15.2)	40.3 (7.7)
Female, n (%)	4 (50.0)	3 (50.0)	2 (33.3)	3 (50.0)	5 (83.3)
Race, n (%)					
White	6 (75.0)	2 (33.3)	6 (100.0)	6 (100.0)	4 (66.7)
Asian	0	1 (16.7)	0	0	1 (16.7)
Black	2 (25.0)	1 (16.7)	0	0	0
Other	0	2 (33.3)	0	0	1 (16.7)
Multiple	0	0	0	0	0
Ethnicity, Hispanic or Latino, n (%)	2 (25.0)	3 (50.0)	1 (16.7)	4 (66.7)	4 (66.7)
Weight, mean (SD), kg	71.0 (7.2)	71.8 (13.6)	76.1 (15.8)	58.0 (5.2)	63.6 (12.2)

MAD cohorts				
Characteristic	Placebo n = 5	Imapextide once weekly		
		10 mg n = 6	30 mg n = 6	30-mg-R*^a^* n = 6
Age, mean (SD), y	47.0 (9.1)	38.3 (8.9)	39.3 (18.3)	36.0 (7.9)
Female, n (%)	0	1 (16.7)	2 (33.3)	2 (33.3)
Race, n (%)				
White	3 (60.0)	6 (100.0)	6 (100.0)	1 (16.7)
Asian	2 (40.0)	0	0	3 (50.0)
Black	0	0	0	1 (16.7)
Native Hawaiian or other Pacific Islander	0	0	0	1 (16.7)
Other or multiple	0	0	0	0
Ethnicity, Hispanic or Latino, n (%)	2 (40.0)	3 (50.0)	2 (33.3)	1 (16.7)
Weight, mean (SD), kg	77.7 (6.9)	77.4 (9.1)	67.6 (4.4)	71.5 (8.0)

DDI cohorts	
Characteristic	Participants n = 14
Age, mean (SD), y	35.9 (12.0)
Female, n (%)	6 (42.9)
Race, n (%)	
White	12 (85.7)
Black	2 (14.3)
Other or multiple	0
Ethnicity, Hispanic or Latino, n (%)	5 (35.7)
Weight, mean (SD), kg	75.6 (11.6)

Abbreviations: DDI, drug-drug interaction; MAD, multiple ascending dose; SAD, single ascending dose.

^*a*^Another cohort received imapextide 30 mg once weekly with medical management of injection site reactions.

### Safety

Imapextide was generally well tolerated. The most common TEAEs in the imapextide treatment groups were injection site erythema (SAD, MAD) and injection site swelling (MAD; Table 2). Among participants who received imapextide, ISRs occurred in 11 of 24 (45.8%) in the SAD cohorts, 17 of 18 (94.4%) in the MAD cohorts, and 13 of 14 (92.9%) in the DDI cohort. The predominant characteristic of ISRs was erythema, with occasional swelling, but no or mild pain, and resolution in approximately 10 days (regardless of the administration of ISR-specific treatments). In 90% of participants who developed ISRs, these events were mild or moderate, localized, and showed no other accompanying general symptoms. Five participants who received imapextide in the MAD cohorts developed larger erythema, which qualified as severe only due to the size (>10 cm; see Supplementary Table S1 (20, 21)), even if there was no associated pain, itching, or systemic inflammatory signs. Of these 5 participants, 1 received a skin biopsy and histological and immunohistochemical assessments showed superficial and deep inflammatory cell infiltration (mainly CD4+ T cells, scattered histiocytes, with few CD8+ T cells, B cells, and mast cells) consistent with a delayed-type hypersensitivity reaction and with no histological (eg, mast cell degranulation) or clinical signs of potential anaphylaxis. Three participants in the MAD cohorts experienced TEAEs (1 in the imapextide 10-mg group [injection site erythema] and 2 in the imapextide 30-mg-R group [1 with injection site erythema, 1 with injection site erythema and injection site swelling]) that led the investigator to withhold administration of the last dose of the study drug. No TEAEs led to study drug discontinuation in the SAD or DDI cohorts. Changes from baseline in vital sign measures, body weight, and electrocardiogram parameters were not clinically significant and did not appear to be dose related (data not shown). All laboratory values for serum chemistry, hematology, urinalysis, and coagulation were generally within the normal range, and none were considered clinically meaningful; there were no laboratory-related TEAEs in any of the 3 study parts. No serious TEAEs or deaths occurred in any cohorts during the study.

**Table 2.** Safety overview for the single ascending dose, multiple ascending dose, and drug-drug interaction cohorts

SAD cohorts					
Parameter, n (%)	Placebo n = 8	Imapextide			
		10 mg n = 6	30 mg n = 6	100 mg n = 6	200 mg n = 6
Any TEAE	4 (50.0)	2 (33.3)	3 (50.0)	3 (50.0)	2 (33.3)
TRAE	0	0	0	1 (16.7)	2 (33.3)
SAE	0	0	0	0	0
TEAE leading to study drug discontinuation	0	0	0	0	0
Most common TEAEs*^a^*					
Injection site erythema	0	0	0	1 (16.7)	2 (33.3)
Dermatitis contact	3 (37.5)	0	1 (16.7)	0	0

MAD cohorts				
Parameter, n (%)	Placebo n = 5	Imapextide weekly		
		10 mg n = 6	30 mg n = 6	30-mg-R*^b^* n = 6
Any TEAE	3 (60.0)	2 (33.3)	5 (83.3)	6 (100.0)
TRAE	1 (20.0)	2 (33.3)	5 (83.3)	6 (100.0)
SAE	0	0	0	0
TEAE leading to study drug discontinuation	0	1 (16.7)*^c^*	0	2 (33.3)*^d^*
Most common TEAEs*^a^*				
Injection site erythema	0	2 (33.3)	3 (50.0)	6 (100.0)
Injection site swelling	0	0	1 (16.7)	3 (50.0)
Hyperglycemia	1 (20.0)	0	0	2 (33.3)
Hypoglycemia	1 (20.0)	1 (16.7)	0	2 (33.3)

DDI cohorts		
Parameter, n (%)	Period 1 (RBX + APAP) n = 14	Period 2 (RBX + APAP + Imapextide 45 mg) n = 14
Any TEAE	2 (14.3)	2 (14.3)
TRAE		
Imapextide-related	0	1 (7.1)
Rosuvastatin-related	0	0
Acetaminophen-related	0	0
SAE	0	0
TEAE leading to study drug discontinuation	0	0

All AEs were coded using the Medical Dictionary of Regulatory Activities v26.1.

Abbreviations: AE, adverse event; APAP, acetaminophen; DDI, drug-drug interaction; MAD, multiple ascending dose; RBX, rosuvastatin; SAD, single ascending dose; SAE, serious adverse event; TEAE, treatment-emergent adverse event; TRAE, treatment-related adverse event.

^*a*^Most common TEAEs are those occurring in 2 or more participants in any treatment group.

^*b*^Another cohort received imapextide 30 mg once weekly with medical management of injection site reactions.

^*c*^One participant with injection site erythema.

^*d*^One participant with injection site erythema and 1 participant with injection site erythema and injection site swelling.

### Pharmacokinetics

**Figure 3.**
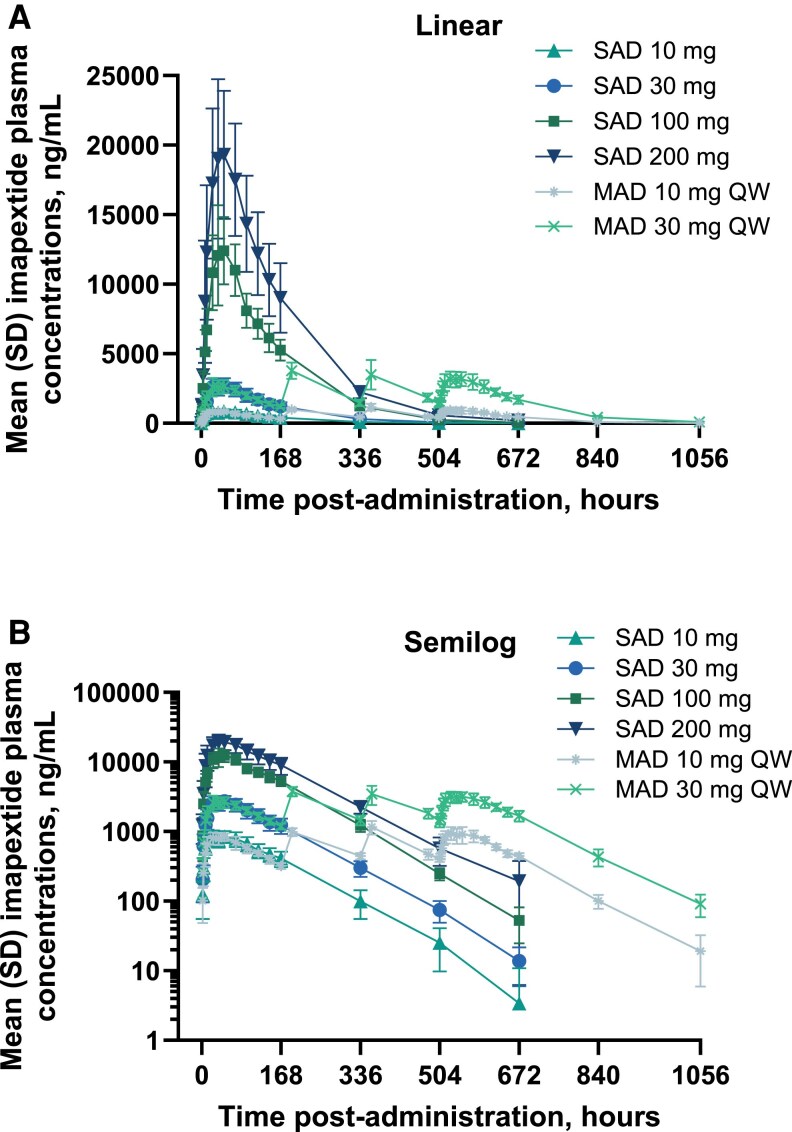
Imapextide concentration-time profiles in the SAD and MAD cohorts. MAD, multiple ascending dose; QW, once weekly; SAD, single ascending dose. Mean (SD) imapextide plasma concentrations presented as A, linear or B, semilog concentration-time curves. Results are reported as observed cases with no imputation for missing data.

Mean imapextide concentration-time profiles in the SAD and MAD cohorts are reported in Fig. 3. In the SAD portion of the study, imapextide concentrations demonstrated a dose-proportional increase over the 10- to 200-mg–dose range (Supplementary Table S2 (21), Supplementary Fig. S1A and S1B (21)). Median t_max_ ranged between 24 to 48 hours, with a trend toward higher values with increasing imapextide doses. In the MAD portion of the study, steady state was achieved approximately 14 days after the first imapextide dose (ie, at the time the third dose was administered; Supplementary Table S2 (21), Supplementary Fig. S1C and S1D (21)). Median t_max_ ranged between 36 and 48 hours at steady state and mean t_1/2_ was approximately 85 hours (∼3.5 days). Minimal accumulation ratio (<1.4 for AUC and C_max_) was observed for the 10- and 30-mg–dose levels, similar to predicted accumulation based on the t_1/2_ and 168-hour (once-weekly) dosing regimen. Exposure parameters for the MAD 30-mg and 30-mg-R cohorts were comparable.

#### Drug-Drug interactions and gastric emptying

**Figure 4.**
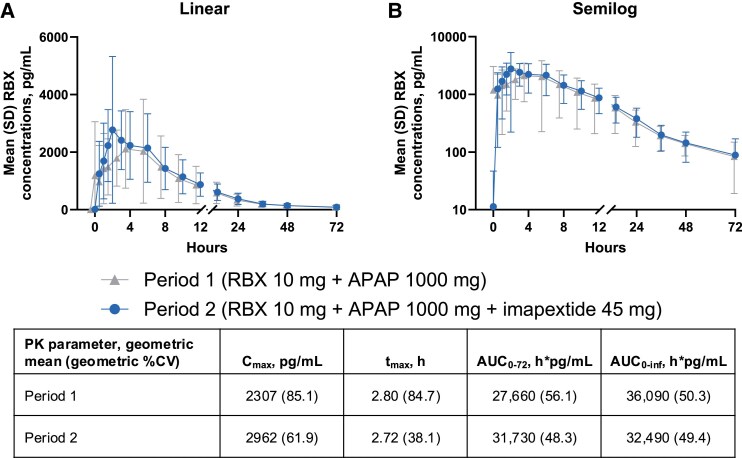
Rosuvastatin concentration-time profiles and PK parameters in the presence or absence of imapextide. APAP, acetaminophen; AUC_0-72_, area under the curve from 0 to 72 hours; AUC_0-inf_, AUC from time 0 extrapolated to infinity; C_max_, maximum plasma concentration; PK, pharmacokinetics; RBX, rosuvastatin; t_max_, time to C_max_. Mean (SD) RBX plasma concentrations presented as A, linear or B, semilog concentration-time curves. Time relative to RBX and APAP administration (study day 1 for period 1 and study day 7 for period 2; imapextide was administered on study day 5). Results are reported as observed cases with no imputation for missing data.

After imapextide administration (study period 2), rosuvastatin plasma concentrations were increased during the absorptive phase (hours 0-4) relative to study period 1, but no differences were identified during the terminal phase (hours 4-72; Fig. 4). A slight increase in rosuvastatin C_max_ (32%), and AUC_0-72_ (22%) was observed in the presence of imapextide compared with rosuvastatin alone, with comparable AUC_0-inf_ and t_max_. Imapextide did not substantially affect CP-I concentrations (AUC and C_max_ differences between treatments of <7%; Supplementary Fig. S2 (21)). Overall, there were no clinically meaningful DDIs.

**Figure 5.**
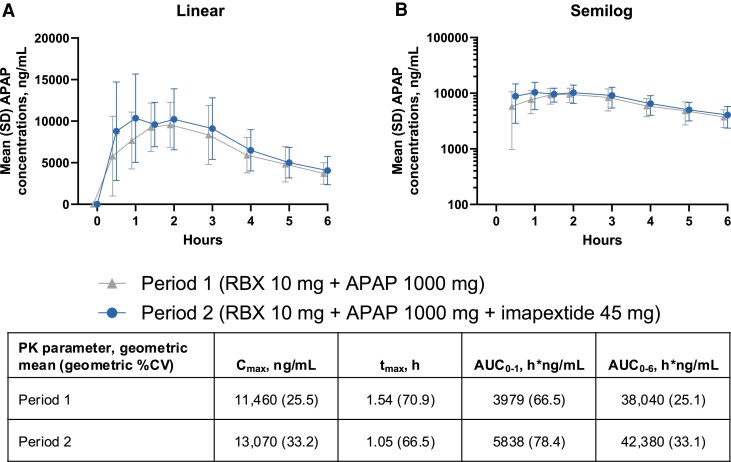
Acetaminophen concentration-time profiles and PK parameters in the presence or absence of imapextide. APAP, acetaminophen; AUC_0-1_, area under the curve from 0 to 1 hour; AUC_0-6_, AUC from 0 to 6 hours; C_max_, maximum plasma concentration; PK, pharmacokinetics; RBX, rosuvastatin; t_max_, time to C_max_. Mean (SD) APAP plasma concentrations presented as A, linear or B, semilog concentration-time curves. Time relative to RBX and APAP administration (study day 1 for period 1 and study day 7 for period 2; imapextide was administered on study day 5). Results are reported as observed cases with no imputation for missing data.

In the presence of imapextide, acetaminophen plasma concentrations were higher at early time points, and acetaminophen C_max_ was modestly increased by 14% relative to acetaminophen alone (Fig. 5). Imapextide appeared to accelerate first-hour gastric emptying (increased acetaminophen AUC_0-1_ by 47%), while overall gastric emptying (AUC_0-6_) was not substantially affected by imapextide treatment.

### Pharmacodynamics

#### Mixed meal tolerance test

**Figure 6.**
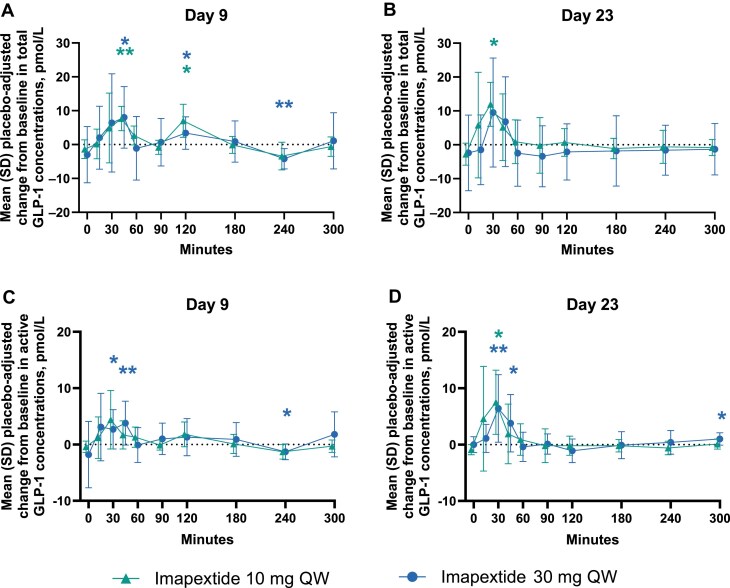
Placebo-adjusted change from baseline in total and active GLP-1 plasma concentrations during an MMTT. GLP-1, glucagon-like peptide-1; MMTT, mixed meal tolerance test; QW, once weekly. *P less than .05; **P less than .01 vs placebo. Mean (SD) placebo-adjusted change from baseline during an MMTT in total GLP-1 concentrations on A, day 9 and B, day 23, and active GLP-1 concentrations on C, day 9 and D, day 23. Results are reported as observed cases with no imputation for missing data.

Placebo-adjusted change from baseline in total GLP-1 levels in the MAD imapextide 10-mg group and the combined 30-mg and 30-mg-R groups was significantly increased during the first 60 minutes of an MMTT on day 9 (1 day after the second dose of imapextide; *P* < .05); similar increases were observed on day 23 (1 day after the fourth dose; Fig. 6A and 6B; Supplementary Fig. S3A and S3B (21)). Significantly increased active GLP-1 levels were also observed at early MMTT time points (30 and 45 minutes; *P* < .05) on days 9 and 23 in the MAD imapextide treatment groups compared with placebo (Fig. 6C and 6D; Supplementary Fig. S3C and S3D (21)). Statistically significant reductions in plasma glucose and increases in C-peptide and insulin concentrations were observed at select postprandial time points with imapextide compared with placebo during the MMTT; however, the magnitude and consistency of these effects varied across time points and dose groups (Supplementary Figs. S4-S6 (21)). No consistent trends in the mean plasma glucagon, plasma PYY, or total GIP concentrations were observed post meal across treatment cohorts or visits.

### Immunogenicity

Treatment-emergent ADAs were observed in 6 of 24 (25.0%) imapextide-treated participants in the SAD cohorts and 11 of 18 (61.1%) imapextide-treated participants in the MAD cohorts; there was also 1 (20%) participant who received placebo in the MAD cohorts who was confirmed ADA positive during the study. ADA-positive responses were of low titer, no dilution, or first dilution (≤300) throughout the study, with only 1 positive time point in most cases, and no apparent effect on the PK or safety of imapextide.

## Discussion

In this phase 1 study of HVs, imapextide was generally well tolerated. PK results supported a once-weekly dosing regimen. Steady-state exposure of imapextide during weekly dosing was achieved after the second dose and was dose proportional over the dose range evaluated. Imapextide had minimal DDIs with rosuvastatin. Furthermore, there was slightly accelerated gastric emptying with imapextide, which is consistent with the known effects of GLP-1 antagonism on gastric motility (22).

ISRs are commonly observed with injectable peptides, including GLP-1 therapeutics. ISRs are a local phenomenon that can comprise a variety of symptoms, including erythema, swelling, pruritus, and pain at and around the injection site (23-25). Although ISRs were reported during the study, they caused minimal discomfort to the participants and were isolated, benign in nature, and manageable. The ISRs were characterized almost exclusively by redness at the site of injection with no or minimal associated pain, tenderness, or itching, and generally resolved within 10 days (with or without treatment). The overall clinical presentation and histological characterization of an ISR skin biopsy from 1 participant were consistent with a delayed-type hypersensitivity reaction, limited to the site of injection, and with no systemic involvement or sign of anaphylactic potential. Importantly, no association between ADAs and ISRs was identified. Taken together, the ISRs observed in this study are unlikely to limit the clinical benefit of imapextide and do not prevent further clinical development. Additional evaluation of ISRs will be conducted in future imapextide studies with longer treatment duration to confirm and support a benign and manageable safety profile.

Despite the small sample size and the study not being powered to detect statistically significant differences, during early time points of an MMTT, imapextide treatment was associated with a significant increase in total and active GLP-1 levels. These apparent effects on GLP-1 may be explained by imapextide competing at the GLP-1 receptor, leading to decreased GLP-1 receptor–mediated clearance and increased circulating GLP-1 concentrations. The current findings are consistent with results from a study showing that avexitide (another GLP-1 receptor antagonist) increased GLP-1 levels in HVs (26) and supports the conclusions that imapextide affects the primary factor contributing to PBH pathophysiology (ie, excessive activation of the GLP-1 receptor caused by exaggerated GLP-1 secretion). Given that imapextide was administered to HVs, the lack of a consistent and conclusive effect in increasing glucose and decreasing insulin peak during an MMTT was expected; similar results were observed with avexitide treatment in HVs (26). Given the rapidly growing prevalence of obesity and the increasing number of bariatric procedures performed worldwide, greater awareness of PBH and advanced therapeutic options are needed. The present study confirms the potential for imapextide to affect a key pathophysiological mediator of PBH, that is, excessive GLP-1 signaling, laying the foundation to evaluate whether imapextide can have a therapeutic benefit of decreasing hypoglycemia in patients with PBH in upcoming studies.

The effects of steady-state exposures of imapextide on rosuvastatin PK parameters (slightly increased C_max_ and AUC_0-72_) suggest that imapextide is either a weak hepatic OATP1B inhibitor or that accelerated gastric emptying affects rosuvastatin's early drug exposure. However, in the presence of imapextide, rosuvastatin exposure was increased only during the absorptive phase (not during the terminal phase). Also, there was no change in CP-I exposure in the presence of imapextide, indicating no pharmacologically relevant inhibition of OATP1B1/3. The increase in acetaminophen C_max_ and AUC_0-1_ in the presence of imapextide suggests that imapextide accelerates gastric emptying. The acetaminophen profile is supportive of an expedited release of rosuvastatin at early time points (without affecting CP-I concentrations), which drives the higher C_max_ without affecting overall AUC_0-inf_. Together, these results suggest that the observed effect of imapextide on rosuvastatin exposure parameters is likely related to accelerated gastric emptying rather than inhibition of OATP1B and suggest minimal potential for clinically relevant DDIs with medications frequently administered to patients who underwent bariatric surgery. The slight increase in first-hour gastric emptying observed with imapextide in a population of HVs could hypothetically affect postprandial hypoglycemia negatively in postbariatric patients. However, these findings from HVs may not be generalizable to patients with PBH, particularly following Roux-en-Y gastric bypass where the stomach and GI tract anatomy have been significantly modified and there is rapid gastric emptying independent and unaffected by imapextide (1, 7). Additionally, any theoretical effect that accelerated gastric emptying may have on hypoglycemic episodes is likely to be counteracted by the inhibitory effect that imapextide is expected to have on exaggerated GLP-1–mediated insulin secretion. In a phase 1 study of patients without diabetes and after Roux-en-Y gastric bypass, gastric emptying of a radiolabeled solid meal was accelerated following avexitide treatment compared with the control group (saline infusion) in the first 45 minutes following food ingestion (27). Additional evaluation of the effect of GLP-1 receptor inhibition on the rate of gastric emptying or GI transit, and other aspects of PBH, is warranted. Future investigations to further characterize the safety and efficacy profile of imapextide in patients with PBH are forthcoming.

Suppression of signaling through the GLP-1 receptor has been shown to prevent postprandial hypoglycemia in patients with PBH and reduce the occurrence of PBH-related symptoms (14-17). The GLP-1 receptor antagonist avexitide significantly reduced the occurrence of hypoglycemia and improved multiple clinical and metabolic parameters with once- or twice-daily treatment in a 28-day, phase 2 study of patients with severe PBH (17). Avexitide 30 mg twice daily demonstrated lower but more consistent exposures (ie, lower peak-to-trough) than 60-mg once-daily dosing. Single dosing daily at 60 mg resulted in a higher tendency for “breakthrough” hypoglycemia in patients putatively due to subtherapeutic concentrations at the end of the dosing interval (17). Based on continuous glucose monitoring data, the mean number of hypoglycemia events was reduced with avexitide vs placebo during daytime hours (8 Am-12 Am); however, this effect was not seen during nighttime hours (12 Am-8 Am), even with twice-daily treatment (17). In assessing this temporal difference in hypoglycemia, it is important to note that the evaluation of nocturnal hypoglycemia with continuous glucose monitoring devices may be unreliable (28). Additionally, there was a reduction in the proportion of participants experiencing hypoglycemia; however, 24% and 12% of patients receiving avexitide 30 mg or 60 mg, respectively, required rescue therapy for hypoglycemia (17), which may be driven by the lack of 24-hour coverage with avexitide. These results suggest that the short half-life of avexitide limits the potential for 24-hour coverage and that higher doses and more prolonged exposure of avexitide would be needed for greater clinical improvements (17). With weekly dosing, imapextide achieved steady-state exposure approximately 14 days after the first dose, with median t_max_ reached within 36 to 48 hours post dose at steady state and minimal imapextide accumulation after 4 weeks of dosing. Furthermore, in vitro data demonstrated that imapextide is up to 9 times more potent than avexitide (Supplementary Table S3 (21)). Taken together, these findings show that the once-weekly dosing regimen of imapextide has the potential to provide sustained PD effects with protection from hypoglycemia through each 24-hour period, including overnight, while reducing injection frequency and improving patient convenience and quality of life.

Limitations of this study include small sample sizes and short dosing duration; however, such designs are typical for phase 1 studies. Most study participants were White, and most participants in the MAD cohort were male. As an exploratory study, no formal statistical comparisons between treatment groups were made due to the small sample size, and apparent differences between treatment groups should be interpreted with caution. Additionally, findings in HVs may be limited by self-selection bias and may not be generalizable to the population of patients with PBH; future studies to evaluate the efficacy and safety of imapextide in patients with PBH have been initiated.

In conclusion, imapextide was generally well tolerated in HVs. The PK results support a weekly dosing regimen, while the PD results show an apparent effect on GLP-1 levels consistent with imapextide potential role in modulating PBH's pathophysiology. Additionally, there were no clinically relevant DDIs with rosuvastatin. These findings support further clinical evaluation of imapextide as a treatment for PBH, aiming to address the unmet need for therapeutic options for patients with PBH.

## Data Availability

MBX Biosciences is committed to providing access to deidentified, patient-level clinical trial data; study reports; study protocols; and statistical analysis plans to qualified researchers on reasonable request. Requests for study data may be submitted via email to info@mbxbio.com. Requesters should include a description of how the data will be used in their research, their plans for disseminating the data in the medical literature, and details about their research team. MBX Biosciences retains the right to approve or reject any request at its sole discretion. If approved, the requestor will be required to enter into a data use agreement with MBX Biosciences. Data requests will be considered for up to 12 months from the publication date of this manuscript.

## Clinical Trial Information

Clinical trial registration number NCT06036784; https://clinicaltrials.gov/study/NCT06036784 (registered August 28, 2023).

## Abbreviations

ADAs: antidrug antibodies
AE: adverse event
APAP: acetaminophen
AUC: area under the plasma concentration-time curve
AUC_0–168_: AUC to the end of the dosing interval
AUC_0–inf_: AUC from time 0 extrapolated to infinity
AUC_0–72_: AUC from 0 to 72 hours
AUC_0–108_: AUC from 0 to 108 hours
AUC_0–1_: AUC from 0 to 1 hour
AUC_0–6_: AUC from 0 to 6 hours
C_max_: maximum plasma concentration
CP-I: coproporphyrin-I
DDI: drug-drug interaction
EC_50_: half-maximal effective concentration
GI: gastrointestinal
GIP: gastric inhibitory peptide
GLP-1: glucagon-like peptide-1
HV: healthy volunteer
IC_50_: half-maximal inhibitory concentration
ISR: injection site reaction
MAD: multiple ascending dose
MMTT: mixed meal tolerance test
PBH: postbariatric hypoglycemia
PD: pharmacodynamics
PK: pharmacokinetics
PYY: peptide YY
RBX: rosuvastatin
SAD: single ascending dose
SAE: serious adverse event
t_1/2_: half-life
TEAE: treatment-emergent adverse event
t_max_: time-to-maximal plasma concentration
TRAE: treatment-related adverse event
